# Supplementary material for: A Critical View of the Application of the APEX Software (Aqueous Photochemistry of Environmentally-Occurring Xenobiotics) to Predict Photoreaction Kinetics in Surface Freshwaters
Source: Molecules. 2019 Dec 18;25(1):9. doi: 10.3390/molecules25010009 (PMC7017383; doi:10.3390/molecules25010009)
Supplement: Supplementary file 1 [file molecules-25-00009-s001.zip › 3.2.4_gcc-4.4.0.zip!/3.2.4_gcc-4.4.0/3.2.4_gcc-4.4.0/doc/octave/html/interpreter/Catching-Errors.html]

Catching Errors - Untitled


Previous: Raising Errors,
Up: Handling Errors


---

#### 12.1.2 Catching Errors

When an error occurs, it can be detected and handled using the
`try` statement as described in The `try` Statement.
As an example, the following piece of code counts the number of errors
that occurs during a `for` loop.

```
     number_of_errors = 0;
     for n = 1:100
       try
         ...
       catch
         number_of_errors++;
       end_try_catch
     endfor
```

The above example treats all errors the same. In many situations it
can however be necessary to discriminate between errors, and take
different actions depending on the error. The `lasterror`
function returns a structure containing information about the last
error that occurred. As an example, the code above could be changed
to count the number of errors related to the \* operator.

```
     number_of_errors = 0;
     for n = 1:100
       try
         ...
       catch
         msg = lasterror.message;
         if (strfind (msg, "operator *"))
           number_of_errors++;
         endif
       end_try_catch
     endfor
```

— Built-in Function: err = **lasterror** (err)  
— Built-in Function: **lasterror** ('reset')  
> Returns or sets the last error message. Called without any arguments
> returns a structure containing the last error message, as well as other
> information related to this error. The elements of this structure are:
>
> 'message': The text of the last error message 'identifier': The message identifier of this error message 'stack': A structure containing information on where the message occurred. This might be an empty structure if this in the case where this information cannot be obtained. The fields of this structure are: 'file': The name of the file where the error occurred 'name': The name of function in which the error occurred 'line': The line number at which the error occurred 'column': An optional field with the column number at which the error occurred
>
> The err structure may also be passed to `lasterror` to set the
> information about the last error. The only constraint on err in that
> case is that it is a scalar structure. Any fields of err that match
> the above are set to the value passed in err, while other fields are
> set to their default values.
>
> If `lasterror` is called with the argument 'reset', all values take
> their default values.

— Built-in Function: [msg, msgid] = **lasterr** (msg, msgid)  
> Without any arguments, return the last error message. With one
> argument, set the last error message to msg. With two arguments,
> also set the last message identifier.

When an error has been handled it is possible to raise it again. This
can be useful when an error needs to be detected, but the program should
still abort. This is possible using the `rethrow` function. The
previous example can now be changed to count the number of errors
related to the \* operator, but still abort if another kind of
error occurs.

```
     number_of_errors = 0;
     for n = 1:100
       try
         ...
       catch
         msg = lasterror.message;
         if (strfind (msg, "operator *"))
           number_of_errors++;
         else
           rethrow (lasterror);
         endif
       end_try_catch
     endfor
```

— Built-in Function: **rethrow** (err)  
> Reissues a previous error as defined by err. err is a structure
> that must contain at least the 'message' and 'identifier' fields. err
> can also contain a field 'stack' that gives information on the assumed
> location of the error. Typically err is returned from
> `lasterror`.
>
>
>
> **See also:** lasterror, lasterr, error.


— Built-in Function: err = **errno** ()  
— Built-in Function: err = **errno** (val)  
— Built-in Function: err = **errno** (name)  
> Return the current value of the system-dependent variable errno,
> set its value to val and return the previous value, or return
> the named error code given name as a character string, or -1
> if name is not found.

— Built-in Function: **errno\_list** ()  
> Return a structure containing the system-dependent errno values.
